# Arogyashreni: towards creating a replicable model for community monitoring of primary health centers in Karnataka, India

**DOI:** 10.1186/1753-6561-6-S5-O25

**Published:** 2012-09-28

**Authors:** Sham N Kashyap, Ramaswami Balasubramaniam

**Affiliations:** 1 Grassroots Research And Advocacy Movement (GRAAM), an initiative of Swami Vivekanada Youth Movement (SVYM), Mysore, India

## Introduction

The National Rural Health Mission (NRHM) has initiated community-based institutions at various levels of the health system e.g. Arogya Raksha Samithi (ARS) and Planning and Monitoring Committee (PMC) at Primary Health Center (PHC) level while the Village Health and Sanitation Committee (VHSC) at the revenue village level. These institutions are intended to create awareness about health services, and to empower communities to demand their entitlements. They are also responsible for planning and monitoring of public health and sanitation programs based on local needs.

However, some barriers including the asymmetry of information and power among health service personnel and community representatives, inefficient decentralization mechanisms, and excessive bureaucracy hinder the effectiveness of these committees. Thus, the Karnataka experience towards communitization has unfortunately been ambivalent.

In this context, an innovative community-based monitoring initiative was devised with objectives of (1) making community-based monitoring operational; (2) developing a sense of ownership among community representatives about their PHCs; (3) bridging information gaps among community representatives to help them articulate local problems and begin to look for their solutions; (4) enhancing demand for health services through community engagement; and (5) building a low-cost technology based platform for rapid analysis and dissemination of information generated through community-based monitoring of health services.

## Methods

This innovative community-based monitoring of health services initiative has been implemented in all rural PHCs of the Mysore district. A questionnaire with close-ended questions (yes/no type) was devised through a community consultation attended by medical doctors, academicians, and community representatives. This questionnaire containing 79 questions on various aspects of PHC functioning was then installed on a toll-free Interactive Voice Response System (IVRS). Selected VHSC and ARS members were oriented to answer the IVRS-based questionnaire. Any selected community member could answer the questionnaire for their respective PHC.

Responses generated through IVRS-based questionnaire were validated through physical verification and statistical assessment of error. Using this base-line community-based monitoring database, a district-wide ranking of rural PHCs was carried out. The ranks were disseminated among the community as well as to the Department of Health and Family Welfare.

## Results

Community representatives responded to the IVRS-based monitoring questionnaire for three consecutive months. Although the medical officers are apprehensive about the community-based monitoring of their PHCs, they concur with the ranks and positions of individual PHCs.

Community representatives are interested in monitoring of health services and in working with the PHC staff, provided that the suggested changes are visible to them. Preliminary analysis of the community’s responses suggests that presence of good physical infrastructure does not necessarily translate into better service delivery. While the T Narasipura block has the highest density of PHCs is the Mysore district, it ranks lowest among all the blocks in the Mysore district in terms of overall utility of the PHCs.

In two of the PHCs, changes brought due to community monitoring have been documented. The district health officer is interested in using the community-based monitoring information as a tool for reviewing PHC progress under NRHM.

## Discussion

This project validates the view that community-based monitoring of health services can be an authentic tool for measuring performance of PHCs and opens up the possibilities to replicate such experiments for other grassroots government institutions as well. The project demonstrates the use of low-cost technology in minimizing data entry/integration issues that adversely affect usability of existing community-based monitoring mechanisms.

The selection of members of VHSCs and ARS for the project was made with the intention to encourage them to bring improvements wherever they can in their role as members of such committees. While positive indications of progress exist, it is too early to conclude that this form of monitoring can also bring sustainable improvements in service delivery at PHCs.

The community-based monitoring and ranking system reports the situation, as it is, without blaming anyone for it. This allowed the monitoring process to evolve into a joint assessment, rather than a community led inspection of the PHC.

Based on these encouraging outcomes, the project aims to target the planning and implementation of activities to be carried out at the PHC level. For this reason, the PMC members shall participate as respondents to the community-based monitoring questionnaire. The advocacy activities of the project will be more focused on need based planning and guaranteeing service delivery in the public health system.

## Competing interests

None declared.

## Funding statement

None declared.

